# Analysis of Stimulant Prescriptions and Drug-Related Poisoning Risk Among Persons Receiving Buprenorphine Treatment for Opioid Use Disorder

**DOI:** 10.1001/jamanetworkopen.2022.11634

**Published:** 2022-05-11

**Authors:** Carrie M. Mintz, Kevin Y. Xu, Ned J. Presnall, Sarah M. Hartz, Frances R. Levin, Jeffrey F. Scherrer, Laura J. Bierut, Richard A. Grucza

**Affiliations:** 1 Department of Psychiatry, Washington University School of Medicine, St Louis, Missouri; 2 Department of Social Work, Washington University in St Louis, St Louis, Missouri; 3 Department of Psychiatry, College of Physicians and Surgeons of Columbia University, New York, New York; 4 Division of Substance Use Disorders, New York State Psychiatric Institute, New York, New York; 5 Department of Family and Community Medicine, St Louis University, St. Louis, Missouri; 6 Department of Health and Outcomes Research, St. Louis University, St Louis, Missouri

## Abstract

**Importance:**

Stimulant medication use is common among individuals receiving buprenorphine for opioid use disorder (OUD). Associations between prescription stimulant use and treatment outcomes in this population have been understudied.

**Objectives:**

To investigate whether use of prescription stimulants was associated with (1) drug-related poisoning and (2) buprenorphine treatment retention.

**Design, Setting, and Participants:**

This retrospective, recurrent-event cohort study with a case-crossover design used a secondary analysis of administrative claims data from IBM MarketScan Commercial and Multi-State Medicaid databases from January 1, 2006, to December 31, 2016. Primary analyses were conducted from March 1 through August 31, 2021. Individuals aged 12 to 64 years with an OUD diagnosis and prescribed buprenorphine who experienced at least 1 drug-related poisoning were included in the analysis. Unit of observation was the person-day.

**Exposures:**

Days of active stimulant prescriptions.

**Main Outcomes and Measures:**

Primary outcomes were drug-related poisoning and buprenorphine treatment retention. Drug-related poisonings were defined using *International Classification of Diseases, Ninth Revision*, and *International Statistical Classification of Diseases and Related Health Problems, Tenth Revision*, codes; treatment retention was defined by continuous treatment claims until a 45-day gap was observed.

**Results:**

There were 13 778 567 person-days of observation time among 22 946 individuals (mean [SD] age, 32.8 [11.8] years; 50.3% men) who experienced a drug-related poisoning. Stimulant treatment days were associated with 19% increased odds of drug-related poisoning (odds ratio [OR], 1.19 [95% CI, 1.06-1.34]) compared with nontreatment days; buprenorphine treatment days were associated with 38% decreased odds of poisoning (OR, 0.62 [95% CI, 0.59-0.65]). There were no significant interaction effects between use of stimulants and buprenorphine. Stimulant treatment days were associated with decreased odds of attrition from buprenorphine treatment (OR, 0.64 [95% CI, 0.59-0.70]), indicating that stimulants were associated with 36% longer mean exposure to buprenorphine and its concomitant protection.

**Conclusions and Relevance:**

Among persons with OUD, use of prescription stimulants was associated with a modest increase in per-day risk of drug-related poisoning, but this risk was offset by the association between stimulant use and improved retention to buprenorphine treatment, which is associated with protection against overdose.

## Key Points

**Question:**

How are use of prescription stimulants associated with treatment outcomes in persons with opioid use disorder (OUD)?

**Findings:**

In this cohort study of 22 946 persons with OUD receiving buprenorphine treatment, stimulant treatment days were associated with 19% increased odds of drug-related poisoning and 36% decreased risk of attrition from buprenorphine treatment.

**Meaning:**

These findings suggest that among persons with OUD who have experienced a drug-related poisoning, stimulant use is associated with a modest increased risk of drug-related poisoning that may be offset by longer duration of buprenorphine treatment, which is associated with protection against overdose.

## Teaser

This cohort study with a case-crossover analytical design examines the associations between prescription stimulant use and drug-related poisonings and buprenorphine treatment retention among patients with opioid use disorder.

## Introduction

Now in its third decade, the current opioid epidemic has claimed an unprecedented number of US lives: More than 500 000 persons have died due to opioid-related overdoses since 1999.^1,2^ Although opioid use disorder (OUD) confers elevated risks of morbidity and mortality,^3,4,5,6^ persons with OUD frequently have co-occurring conditions that may increase these risks even further. For example, attention-deficit/hyperactivity disorder (ADHD) is estimated to occur in 20% to 25% of persons in who seek treatment for OUD,^7,8,9^ and co-occurring ADHD portents worse substance use disorder (SUD)–related outcomes, including lower prevalence of SUD treatment participation^10^ and higher rates of treatment attrition.^11^ Depression is also common among persons with OUD: As many as 48% of persons with OUD have met criteria for a lifetime major depressive episode,^12^ and persons with OUD have an elevated risk of suicide.^3,13^ Finally, persons with OUD have become increasingly likely to use psychostimulants—particularly methamphetamine—over time, perhaps to combat the sedating effects of increasingly potent illicitly made opioids such as fentanyl.^14^ For example, the percentage of persons in OUD treatment who also reported using methamphetamine increased from 2% to almost 13% from 2008 to 2017.^15^ Disturbingly, the number of overdose deaths involving both opioids and stimulants has increased dramatically in recent years,^16,17,18^ and fatalities due to this coingestion are often referred to as the “fourth wave” of the opioid epidemic.^19^ Thus, identifying optimal treatments for these particularly high-risk subgroups of persons with OUD is critical to help prevent subsequent opioid-related deaths.

Medication treatment is considered standard of care for OUD.^20^ Of the 3 medications approved by the US Food and Drug Administration for OUD, buprenorphine, a partial opioid agonist, is most commonly prescribed^21,22^ and has been repeatedly shown to be effective for relapse prevention^23,24^ and to be associated with decreased risk of overdose and death.^4,25,26,27,28^

Centrally acting stimulants carry US Food and Drug Administration approval for ADHD treatment in both children and adults and are considered first-line treatments for ADHD in these populations.^29,30^ In addition to ADHD, stimulants are commonly used off-label to treat other conditions, including depression and stimulant use disorders.^31^ In fact, as many as 50% of stimulant prescriptions may be written for off-label conditions in adults,^32,33^ and the prevalence of stimulant prescriptions has increased over time, particularly among adults.^34^

Because stimulants carry the potential for misuse,^35,36,37^ clinicians may be reluctant to prescribe them to persons with co-occurring SUD owing to concern that the stimulants may increase the risk of SUD relapse.^38^ Although existing data to provide definitive guidance on this issue are lacking,^39^ a 2017 study using insurance claims data found that stimulant prescriptions were associated with decreased risk of emergency department visits related to substance use among persons with an SUD diagnosis.^40^ Further, several randomized clinical trials have examined stimulant use in adults with co-occurring stimulant use disorder and ADHD and found either a lack of association between stimulant prescriptions and illicit drug use^41^ or a protective effect.^42,43^

Outcomes among persons who are prescribed stimulants and have co-occurring OUD, however, are particularly understudied. We know of only 2 prior studies^44,45^ that have examined stimulant use in populations with OUD. Both studies focused on persons in methadone maintenance programs who had co-occurring ADHD diagnoses and were treated with stimulants, and neither study found an association between stimulant use and illicit drug use risk,^44,45^ although generalizability of results is limited by strict inclusion criteria, small sample sizes, and short follow-up periods. We know of no previous studies that have examined associations between stimulant use and SUD-related outcomes in persons with OUD treated with buprenorphine.

In addition to illicit drug use, treatment retention is increasingly considered a clinically meaningful outcome in SUD research.^46,47^ For persons with OUD, longer treatment retention has been associated with lower overdose risk.^25^ Recent evidence indicates that stimulant treatment may improve SUD treatment retention among persons with co-occurring SUD and ADHD^48^; thus, whether prescription stimulant use is associated with improved buprenorphine treatment retention in persons with OUD is an important clinical question.

Observational cohort studies may be particularly helpful for addressing questions about prescription stimulant medication use and OUD outcomes given their allowance for large sample sizes, real-world populations, and potential for relatively long follow-up periods. Thus, in this study, we used large insurance claims databases including persons with OUD receiving buprenorphine treatment and a case-crossover analytical design to examine associations between prescription stimulant use and 2 clinically relevant OUD-related outcomes: (1) drug-related poisonings and (2) buprenorphine treatment retention.

## Methods

### Data Source

This cohort study with a case-crossover design accessed IBM MarketScan Commercial and Multi-State Medicaid databases, which included information from January 1, 2006, to December 31, 2016, on use of health care resources, expenditures, and prescription pharmacy files across both employer-sponsored and Medicaid health plans. Analyses were conducted from March 1 through August 31, 2021. Data were deidentified, thus the study was exempted from human subjects review and informed consent by the Washington University in St Louis institutional review board. The study followed the Strengthening the Reporting of Observational Studies in Epidemiology (STROBE) reporting guideline.

### Participants

**Figure 1.**
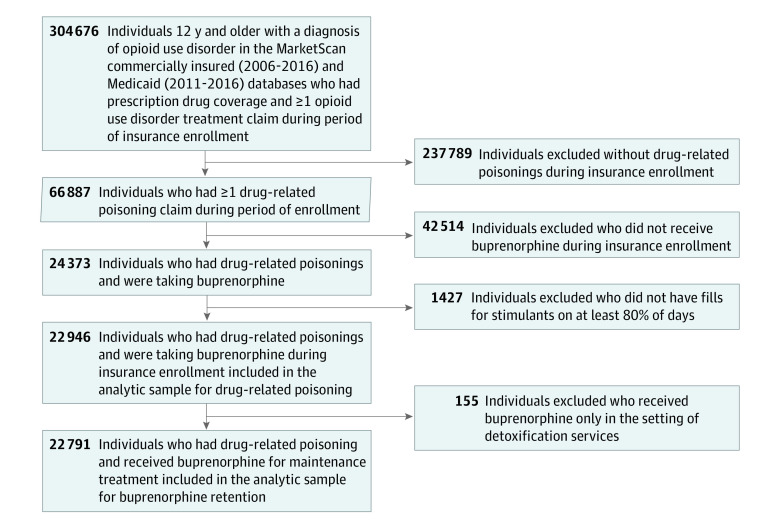
Development of Study Sample

The analytical sample included individuals with OUD aged 12 to 64 years who (1) received a buprenorphine prescription for longer than 1 day and (2) experienced at least 1 drug-related poisoning episode during insurance enrollment. Persons who met inclusion criteria but were not prescribed stimulants were included in analyses to inform covariate estimates. For similar reasons, we included persons who received buprenorphine at some point during their insurance coverage but not necessarily during the study observation period. For buprenorphine treatment retention analyses, because the purpose was to evaluate maintenance OUD treatment outcomes, we excluded the small number of individuals who received buprenorphine only in the setting of detoxification services. Figure 1 depicts the selection of the analytical sample. Additional details regarding derivation of the cohort can be found elsewhere.^22,49,50,51^

### Design

We conducted a within-person, repeated-event cohort study with a case-crossover design. Primary units of observation were person-days. Each person-day was characterized by receipt or absence of a stimulant prescription. For our first objective, case periods represented days on which a drug-related poisoning occurred and were coded as 1; control periods were days when poisonings did not occur and were coded as 0. Information about each participant’s exposure to medication during case periods was compared with the same individual’s exposure distribution during control periods.^52^ An individual’s first observed poisoning event served as the index event, and individuals could be observed for as long as 1 year before and 1 year after this event. We chose a 2-year maximum observation period because this was the mean duration of time participants were in the data set. Participants could experience multiple drug-related poisonings as long as they occurred in the year after the index event; observations in the year before the index event were used to improve covariate estimates. Individuals who lost insurance coverage during the observation window were censored.

For our second objective, the case day was the last day of the buprenorphine treatment episode and was coded as 1; all other days constituted control days and were coded as 0. Treatment days were characterized by the presence or absence of stimulant prescription. There was no maximum observation time imposed for retention analyses, but observation was limited to days within the treatment episode. Individuals who lost insurance coverage during a treatment episode and those who were retained in treatment throughout the duration of their enrollment were censored.

### Primary Outcomes

Our first primary outcome was drug-related poisoning. We defined this outcome as any emergency department visit or inpatient hospitalization involving *International Classification of Diseases, Ninth Revision*, or *International Statistical Classification of Diseases and Related Health Problems, Tenth Revision*, codes for a drug-related poisoning. Methods for identifying these claims have been described previously,^50,51^ and diagnostic classifications are provided in the eTable in the Supplement.

Our second primary outcome was buprenorphine treatment retention. We defined treatment retention as described previously^22,49^: namely, continuous periods of time characterized by receipt of buprenorphine, counseling, or both. We defined treatment episodes by first utilization of buprenorphine or psychosocial treatment and continuing until a gap of at least 45 days without claims for either psychosocial treatment or buprenorphine was encountered. Individuals were permitted to undergo multiple treatment episodes.

Buprenorphine prescriptions were defined using National Drug Codes.^22,49,50,51^ We assumed an active prescription connoted medication consumption. For example, if a participant received a 30-day prescription for buprenorphine, they were assumed to have taken buprenorphine for each of the 30 days for which the prescription was written. To test whether buprenorphine treatment episodes were valid proxies for buprenorphine exposure, we evaluated the percentage of days during a treatment episode after initiating buprenorphine as evidenced by buprenorphine prescriptions and days of supply: 84% of buprenorphine treatment episode days were covered by a buprenorphine prescription, providing support that the treatment episode construct was a valid approximation for buprenorphine exposure. Psychosocial treatment claims were defined using *Current Procedural Terminology* codes.^22,49^

### Ascertainment of Exposures

Our exposure variable comprised stimulant treatment days. Stimulant prescriptions were identified using generic drug names that included the following words: amphetamine, methylphenidate, or lisdexamfetamine. Because we were interested in the associations between our outcomes and stimulants prescribed as maintenance treatment, we excluded persons for whom the mean ratio of stimulant days supplied to days between fills was less than 0.8.

### Covariates

Because each individual serves as their own control in a case-crossover design, time-invariant covariates (eg, demographic information, SUD and non-SUD comorbidities) were not included as covariates. We extracted data on age, sex, race and ethnicity (available in the Medicaid data set only), insurance status, and comorbidities for descriptive purposes.

The repeated-event design allowed us to incorporate time as a covariate.^52,53,54^ We also included benzodiazepines and statins as time-varying covariates in our model estimating drug-related poisoning risk. Benzodiazepines were included given their high prevalence of use in populations with SUD and prior work^50^ demonstrating a statistical interaction between benzodiazepine and buprenorphine exposure when estimating risk for drug-related poisoning. Statins were included in a negative control to evaluate whether our inclusion of time as a covariate was adequate to control for persistent user bias.^50,55^ We included statins as a covariate in our model estimating buprenorphine treatment retention to examine the degree to which healthy adherer bias^56^—that is, that persons who are engaged in treatment for one condition are more likely to engage in treatment for all conditions—might influence any association between stimulants and retention.

### Statistical Analysis

Analyses were conducted using SAS, version 9.4 (SAS Institute Inc). We used a fixed-effect conditional logit model to estimate the risk of drug-related poisoning events as a function of stimulant exposure while controlling for secular time trends and time from index drug-related event using restricted cubic splines. Effect sizes were measured as odds ratios (ORs) that, given the rarity of the primary outcomes, were essentially equal to risk ratios.

We conducted several secondary analyses. First, because both the potency of misused opioids and the number of opioid-related deaths have increased with time,^1,18^ we stratified the sample into 2 periods to examine whether estimates differed by time. We identified the median date within the data set, rounded that date to the nearest calendar month, then estimated drug-related poisoning risk for the first and second halves of the study period. Second, because stimulants are more commonly prescribed for off-label reasons in adult populations,^32^ we stratified our sample by age (12-29 and 30-64 years) to determine whether estimates differed by age group. Finally, we estimated the association between stimulants and drug-related poisoning risk in persons who were prescribed stimulants during their insurance enrollment to evaluate whether results were similar to those obtained for the primary analytical sample. For buprenorphine treatment retention, we used a fixed-effect conditional logit model to estimate risk of a treatment attrition event (the last date of retention) as a function of stimulant exposure during the treatment episode.

## Results

### Sample Characteristics

As shown in Table 1, the analytical sample for drug-related poisoning analyses consisted of 22 946 persons. A total of 11 393 women (49.7%) and 11 553 men (50.3%) were included. The mean (SD) age was 32.8 (11.8) years. A total of 8169 patients (35.6%) had Medicaid insurance. In regard to co-occurring diagnoses, 1154 patients (5.0%) had ADHD, 4383 (19.1%) had a major depressive disorder, and 2553 (11.1%) had a stimulant use disorder in the 6 months before the first OUD treatment claim. A total of 16 180 persons (70.5%) received buprenorphine during the year before or after the index drug-related poisoning event, and 2470 (10.8%) received at least 1 stimulant prescription during this time. The mean (SD) observation time was 600 (145) days.

**Table 1.** Demographic Characteristics of Persons With Opioid Use Disorder Prescribed Buprenorphine During Insurance Enrollment Who Experienced a Drug-Related Poisoning

Characteristic	Patient data (N = 22 946)^a^
Sex	
Men	11 553 (50.3)
Women	11 393 (49.7)
Insurance	
Commercial	14 777 (64.4)
Medicaid	8169 (35.6)
Race and ethnicity^b^	
Black	436 (5.6)
Hispanic	88 (1.1)
White	6098 (78.6)
Other	1135 (14.6)
Age, mean (SD), y	32.8 (11.8)
Co-occurring SUD	
Stimulant^c^	2553 (11.1)
Alcohol	3562 (15.5)
Sedative	2317 (10.1)
Co-occurring non-SUD psychiatric disorder	
Major depressive disorder	4383 (19.1)
Anxiety disorder	8745 (38.1)
Psychotic disorder	901 (3.9)
ADHD	1154 (5.0)
Medication prescription during year before and after index drug-related poisoning	
Buprenorphine	16 180 (70.5)
Stimulant	2470 (10.8)
Benzodiazepine	13 210 (57.6)
Statin	1674 (7.3)

Abbreviations: ADHD, attention-deficit/hyperactivity disorder; SUD, substance use disorder.

^a^ Unless otherwise indicated, data are expressed as number (%) of patients. Totals in mutually exclusive categories may not sum to 100 owing to rounding.

^b^ Available among 7757 Medicaid recipients only for categories as identified.

^c^ Includes persons with diagnosis of cocaine use disorder and/or amphetamine-type use disorder (including methamphetamine).

### Stimulant Medication and Drug-Related Poisoning Risk

Table 2 presents the odds of drug-related poisoning as a function of medication. Stimulant treatment days were associated with a 19% increased odds of drug-related poisoning relative to no treatment (OR, 1.19 [95% CI 1.06-1.34]). As expected, buprenorphine treatment days were associated with a 38% decreased risk of drug-related poisoning relative to no treatment (OR, 0.62 [95% CI, 0.59-0.65]). There were no interactions observed (ie, the effects of stimulants were independent of buprenorphine treatment and vice versa). As anticipated,^50^ benzodiazepines were associated with increased risk of drug-related poisoning (OR, 1.93 [95% CI, 1.84-2.03]); statins were not associated with drug-related poisoning risk (OR, 0.99 [95% CI, 0.86-1.13]). Results from secondary analyses showed similar results, albeit with wider 95% CIs.

**Table 2.** Adjusted Odds of Drug-Related Poisoning Associated With Stimulant Use Among Persons With Opioid Use Disorder Prescribed Buprenorphine

Medication	OR (95% CI)
**Main model^a^**	
Stimulant	1.19 (1.06-1.34)
Buprenorphine	0.62 (0.59-0.65)
Benzodiazepine	1.93 (1.84-2.03)
Statin	0.99 (0.86-1.13)
**Stratified by time** ^b^ ^,^ ^c^	
January 1, 2006, to May 31, 2013	
Stimulant	1.29 (1.09-1.52)
Buprenorphine	0.59 (0.55-0.64)
Benzodiazepine	2.05 (1.92-2.19)
Statin	0.94 (0.79-1.13)
June 1, 2013, to December 31, 2016^d^	
Stimulant	1.13 (0.95-1.35)
Buprenorphine	0.65 (0.60-0.70)
Benzodiazepine	1.78 (1.65-1.92)
Statin	1.00 (0.82-1.23)
**Stratified by age**	
12-29 y^e^	
Stimulant	1.22 (1.04-1.44)
Buprenorphine	0.63 (0.58-0.68)
Benzodiazepine	2.18 (2.01-2.35)
Statin	0.85 (0.42-1.75)
30-64 y^f^	
Stimulant	1.13 (0.94-1.35)
Buprenorphine	0.60 (0.56-0.65)
Benzodiazepine	1.79 (1.68-1.90)
Statin	0.99 (0.87-1.13)
**Stimulant prescription during insurance enrollment** ^g^	
Stimulant	1.19 (1.05-1.34)
Buprenorphine	0.63 (0.56-0.72)
Benzodiazepine	1.96 (1.75-2.19)
Statin	1.09 (0.75-1.58)

Abbreviation: OR, odds ratio.

^a^ Includes 22 946 persons (13 778 567 person-days).

^b^ For time stratification analyses, it was possible that individuals could be counted twice, given the longitudinal nature of data set.

^c^ Includes 13 449 persons (6 886 861 person-days).

^d^ Includes 14 037 persons (6 891 706 person-days).

^e^ Includes 10 977 persons (6 607 490 person-days).

^f^ Includes 11 969 persons (7 117 077 person-days).

^g^ Includes 3628 persons (2 250 582 person-days).

### Stimulant Medication and Buprenorphine Treatment Retention

In total, 22 791 persons and 7 940 667 person-days were included in retention analyses. Stimulant treatment days were associated with 36% decreased odds of buprenorphine treatment attrition (OR, 0.64 [95% CI, 0.59-0.70]); there was no association between statins and buprenorphine treatment retention (OR, 0.96 [95% CI, 0.86-1.07]).

## Discussion

In this study examining associations between stimulant use and treatment outcomes in persons with OUD, we had 2 main findings. First, stimulant treatment days were associated with 19% increased odds of drug-related poisonings. Second, there was an approximately 36% decreased odds of attrition from buprenorphine treatment.

Although the risk of drug-related poisoning was modest, clinicians should be cognizant of this potential safety risk when considering whether to prescribe stimulants to patients with OUD who do not wish to receive buprenorphine. Interpretation of the observed association between prescription stimulant use and drug-related poisoning risk deserve careful consideration.

First, previous studies have shown that prescription stimulants are associated with misuse, even among persons for whom they are prescribed. For example, more than 25% of adolescents who use stimulants for medical reasons also report misusing their prescription.^37^ There is also evidence that almost 20% of college students misuse prescription stimulants^36^ and more than 30% of adults who use stimulants misuse them.^35^ Reasons for misuse are multifactorial and may not necessarily be related to addiction. For example, common reasons for misuse are to improve concentration^35^ or academic performance^57,58^; however, misuse for these purposes can still increase likelihood of a negative outcome.

Second, our sample represents a particularly ill subset of the population with OUD: those with a documented history of drug-related poisoning events. It is a reasonable assumption, then, that our cohort had a higher likelihood of experiencing negative outcomes with prescription stimulants than a population who had not previously had a drug-related poisoning. Whether or not our findings are consistent among a cohort of individuals who have not yet experienced a drug-related poisoning at the start of the study is an important direction for future research.

Finally, it is possible that the association between stimulant use and drug-related poisoning risk differs by the indication for which a stimulant is prescribed. Although stimulants are commonly prescribed for ADHD in adolescents, as many as 50% of stimulant prescriptions are for off-label indications in the adult population.^32^ The wide 95% CIs suggesting lack of statistical power limit any interpretation of potential age differences with respect to stimulant use and drug-related poisoning risk in our results. Whether the association between stimulant use and drug-related poisoning risk is different when the stimulant is prescribed for ADHD vs stimulant use disorder or depressive illness is an important area for future research.

It should be noted that our data provide no insight into the effectiveness of stimulant medications for treating targeted symptoms (eg, impulsivity associated with ADHD, or anhedonia associated with depression), nor do they provide information on the risks associated with untreated symptoms that stimulants may benefit. For example, untreated ADHD has been associated with higher risks of accidents^59^ and suicide.^60^ Clinicians should weigh the risks and benefits of prescribing stimulants thoughtfully when treating persons with OUD and a co-occurring condition that may benefit from stimulant treatment.

Importantly, stimulant use was associated with greater duration of buprenorphine treatment. This finding is consistent with previous work demonstrating stimulants may improve SUD treatment retention.^61^ Buprenorphine is a known protective factor against overdose among persons with OUD,^4,25,26,27^ and in our own data set, buprenorphine was associated with a 38% decreased odds of drug-related poisonings. Given the relatively low retention rates of long-term buprenorphine use,^22,49^ the association between stimulant use and increased likelihood of buprenorphine retention is notable.

**Figure 2.**
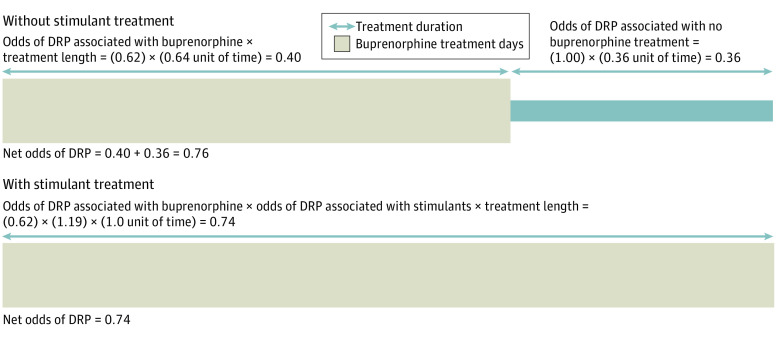
Illustration of the Net Risk of Drug-Related Poisoning Among Persons With OUD Receiving Buprenorphine With and Without Co-use of Prescription Stimulants DRP indicates drug-related poisoning.

Persons prescribed both buprenorphine and stimulants, then, were susceptible to both the risk (increased per-day overdose odds) and protective (increased buprenorphine exposure) associations conferred by stimulants. It is therefore important to weigh the magnitude of both of these associations and how they might be related to net treatment outcomes. Figure 2 provides a visual interpretation of a potential reconciliation of these opposing results. Extrapolating from our result that individuals were 36% less likely to cease buprenorphine treatment when using stimulants, Figure 2 demonstrates that stimulant-involved buprenorphine treatment episodes are approximately 36% longer than treatment episodes for which stimulants are not prescribed. Further, we illustrate that the increased per-day risk of drug-related poisoning associated with stimulants combined with the increased exposure to buprenorphine’s protective effects against overdose results in a net 26% decreased odds of drug-related poisoning relative to no buprenorphine treatment. This protective association is similar in magnitude to the risk associated with receipt of buprenorphine without stimulants, because although the per-day protection associated with buprenorphine without stimulants is stronger (OR, 0.62), we found that length of the treatment episode in the absence of stimulants is shorter, resulting in less buprenorphine exposure over time (Figure 2). However, we cannot rule out certain sources of confounding that may affect our analyses. Regardless, Figure 2 illustrates that both the risks and the benefits of stimulant medication on OUD treatment should be considered when treating persons with co-occurring OUD and conditions for which stimulants may be indicated and provides evidence that when buprenorphine is co-prescribed with stimulants to persons with OUD, they may have comparable protection against overdose over time.

### Limitations

This study has some limitations. Case-crossover designs cannot control for time-varying confounding. We attempted to evaluate for confounding by calendar time by stratifying our sample by time and found effect sizes were in the same direction for each period examined, although wide 95% CIs preclude definitive interpretation. Another form of time-varying confounding can arise if within-person exposure and outcome events are not independent. Although it is impossible to evaluate this assumption, a previous analysis of buprenorphine use in the context of benzodiazepine use^50^ found that effect size estimates were robust to selection of time window relative to drug-related poisoning. It is also important to note that our most recent year of data analyzed was 2016; given that illicitly manufactured fentanyl did not become the main cause of opioid-related deaths until after 2015,^1^ replication of these analyses with more recent data is an important direction of future research. Pharmacy claims do not always reflect actual consumption of medication; however, for both buprenorphine and stimulants, proportion of days covered in a treatment episode was high, suggesting few gaps between refills and providing some evidence of likely consumption. Owing to the nature of insurance claims data, our study focused on drug-related poisonings that resulted in emergency department or hospital admissions, although it should be noted that many drug-related poisonings do not result in health care system contact; therefore, we cannot be certain that the risk associations we found in our sample generalize to poisonings that do not come to medical attention. Further, although most drug-related poisonings are not fatal,^62^ we did not exclude drug-related poisonings that resulted in death during hospitalization from our outcome definition. Finally, residual confounding by unmeasured time invariant variables cannot be ruled out.

## Conclusions

The findings of this recurrent-event cohort study with a case-crossover design suggest that stimulant medication was associated with a modest increased risk of drug-related poisoning among persons with OUD. However, this risk may be offset by the association between stimulant use and buprenorphine treatment retention, resulting in prolonged exposure to buprenorphine, which is associated with protection against overdose.

## Notes

Supplement. **eTable.** Diagnosis Codes for Opioid Use Disorder and Drug-Related Poisonings
eReferences
