# Supplementary material for: Analysis of Stimulant Prescriptions and Drug-Related Poisoning Risk Among Persons Receiving Buprenorphine Treatment for Opioid Use Disorder
Source: JAMA Netw Open. 2022 May 11;5(5):e2211634. doi: 10.1001/jamanetworkopen.2022.11634 (PMC9096599; doi:10.1001/jamanetworkopen.2022.11634)
Supplement: Supplement. — eTable. Diagnosis Codes for Opioid Use Disorder and Drug-Related Poisonings eReferences [file jamanetwopen-e2211634-s001.pdf]

# Supplementary Online Content

Mintz CM, Xu KY, Presnall NJ, et al. Analysis of stimulant prescriptions and drug-related poisoning risk among persons receiving buprenorphine treatment for opioid use disorder. *JAMA Netw Open*. 2022;5(5):e2211634. doi:10.1001/jamanetworkopen.2022.11634

**eTable.** Diagnosis Codes for Opioid Use Disorder and Drug-Related Poisonings
**eReferences**

This supplementary material has been provided by the authors to give readers additional information about their work.

© 2022 Mintz CM et al. *JAMA Network Open*.

**eTable.** Diagnosis Codes for Opioid Use Disorder and Drug-Related Poisonings

| Diagnosis | ICD-9/-10-CM diagnosis codes |
|---|---|
| Opioid Use Disorder | 305.5, 304.0, 304.7, F11 |
| Drug-Related Poisoning | T40, T41, T42, T43, T44, T45, T46, T47, T48, T49, T50, T51, T52, T53, T54, T55, T56, T57, T58, T59, T60, T61, T62, T63, T64, T65<br><br>960, 961, 962, 963, 964, 965, 966, 967, 968, 969, 970, 971, 972, 973, 974, 975, 976, 977, 978, 979, 980, 981, 982, 983, 984, 985, 986, 987, 988, 989<br><br>E850, E851, E852, E853, E854, E855, E856, E857, E858, E860, E861, E862, E863, E864, E865, E866, E867, E868, E869, E950, E951, E952, E962, E972, E975, E976, E980, E981, E982 |

ICD= International Classification of Diseases; CM=Clinical Modification.

For more detailed information, these classifications for opioid use disorder and drug-related poisoning are described in the MarketScan analyses conducted by our group below.

**eReferences**


Mintz, CM, Presnall, NJ, Xu KY, et al. 2021. An examination between treatment type and treatment retention in persons with opioid and co-occurring alcohol use disorders. *Drug and Alcohol Depend.* 10.1016/j.drugalcdep.2021.108886. NIHMSID: NIHMS1726278

Xu KY, Presnall, NJ, Mintz CM et al. 2021. Association of opioid use disorder treatment and alcohol-related acute events. *JAMA Network Open,* 4(2): e210061. PMID: 33625511

Xu KY, Borodovsky JT, Presnall N, et al. Association Between Benzodiazepine or Z-Drug Prescriptions and Drug-Related Poisonings Among Patients Receiving Buprenorphine Maintenance: A Case-Crossover Analysis. *Am J Psychiatry,* 2021:appiajp202020081174. NIHMSID: NIHMS 1673224

Mintz CM, Presnall NJ, Sahrmann JM, et al. 2020. Age disparities in six-month treatment retention for opioid use disorder. *Drug Alcohol Depend.* 13:108130. NIHMSID: NIHMS1662180**.**


© 2022 Mintz CM et al. *JAMA Network Open*.
